# Analysis of the Turnover Tendency of College Teachers From the Perspective of Psychology

**DOI:** 10.3389/fpsyg.2022.771324

**Published:** 2022-06-21

**Authors:** Ying Zhao, Kai Zhou

**Affiliations:** ^1^School of Public Administration, Jilin University, Changchun, China; ^2^School of Cyber Security, Changchun University, Changchun, China

**Keywords:** psychological analysis, university teachers, turnover tendency, regression model, support system

## Abstract

University teachers are the core of university teaching and scientific research construction and an important link of double first-class construction. With the increasingly fierce academic competition among colleges and universities, the resignation behavior of college teachers is increasing, which has brought great impact on the construction of talents and discipline development in colleges and universities. The survey found that the departure of college teachers is not an isolated phenomenon, but a time-consuming process. Although the turnover tendency of college teachers has not evolved into resignation behavior in most cases, the resignation tendency of college teachers is always accompanied by negative psychology, complaints, and perfunctory work. These bad phenomena have brought double negative effects to colleges and universities and teachers themselves. According to this, psychological analysis of the turnover tendency of college teachers is conducive to building a benign flow mechanism for college teachers, thus supporting the long-term development of colleges and universities.

## Introduction

According to the 2016 National Teacher Development Survey (the investigation project organized and implemented by The School of Education of Peking University from 2016 to 2017) data, 66% of teachers have different degrees of turnover tendency (Du and Liu, 2019). At present, some domestic universities try to rapidly improve their own teachers’ level and strengthen and consolidate the dominant learning through large-scale talent introduction measures with high incentives as the core division status so as to strengthen and consolidate their position as superior disciplines. However, this kind of talent introduction measure, which is basically oriented by economic interests, has brought problems such as excessive talent cost, and emphasis on quantity over quality. Even cases of talents imported with heavy funds are difficult to carry out their work due to insufficient hardware facilities. The resignation tendency of college teachers has been paid attention to by the academic community earlier and has now formed certain results. Taken together, these results are mainly focused on two levels. First, the psychological mechanism of college teachers’ tendency to leave; Du and Liu (2019) analyzed the internal relationship between the perception of academic power and the tendency of college teachers to leave through investigation and research. Yin Muzi paid attention to the new college teachers and interpreted in detail the huge pressure of college teachers under the pressure of scientific research and the mechanism and development path of their resignation tendency. Zhao Chonglian and Zheng Yong analyzed the emergence and evolution of college teachers’ turnover tendency from the perspective of job burnout. Second, the coping strategies of college teachers’ tendency to leave. Zhu Naiping and Jiang Dan paid attention to the over construction of college teachers’ influencing factor model of college teachers’ resignation tendency and discussed the relationship between career pressure, performance appraisal, job satisfaction, and organizational commitment on resignation tendency. Cao Yuping paid attention to the separation process and cost analysis of college teachers and recorded the design of the response plan in the process of analyzing the turnover tendency of college teachers. The above results have had a certain impact on this study. At the same time, an important innovation point of this study is to adopt data quantification, analyze qualitative research plans, and then produce more objective results.

## Psychology and Explaining the Tendency of College Teachers to Leave

### Mechanism of the Resignation Tendency of College Teachers

The tendency to leave means that the organization has lost its attraction to employees, causing employees to have attitudes and ideas to leave the organization. The turnover tendency of college teachers is a process phenomenon (Pei, 2004). In the beginning, the identity of the organizational culture decreased or disappeared, accompanied by gradual negative work attitude, which eventually evolved into a conflict with the organization at the conceptual level, and finally became more negative or divorced from the organization. The turnover tendency of college teachers is an interaction between individual decision-making and the external environment provided by colleges and universities. The external environment provided by colleges and universities is an important trade-off factor for college teachers to make resignation decisions. College teachers are faced with the influence of the external environment, which is the core of school treatment and assessment index. Generally speaking, the turnover tendency of university teachers is an interaction between individual decision-making and the external environment provided by the university. When individuals think that the external environment provided by the school cannot meet their psychological needs, they often have the tendency to leave their jobs.

The resignation of college teachers is an objective practical problem, but the resignation of colleges and universities and teachers is not an isolated phenomenon. College teachers generally have a “critical period” of 6 months from the tendency to resignation behavior. Starting from the “critical period,” we can establish an effective mechanism to protect the right of teachers’ turnover by recognizing and understanding the internal mechanism of teachers’ turnover from the perspective of psychology. On the one hand, the right of teachers’ turnover can be guaranteed, and on the other hand, the development of colleges and universities can be guaranteed with sufficient intellectual support. Therefore, psychological analysis of the turnover tendency of college teachers can put forward corresponding countermeasures to alleviate the turnover tendency of college teachers in a critical period of time, and then stabilize the ranks of college teachers.

### Psychological Overview of College Teachers’ Tendency to Leave

This study, when discussing the turnover tendency of college teachers, comprehensively draws on the basic demand theory, psychological contract theory, and the relevant theory of “cognitive psychology” in psychology, and then integrates and analyzes the basic perspective of college teachers’ turnover tendency.

The basic psychological needs theory was put forward by Deci and Ryan (2000). It elaborates the mechanism theory of the role of environmental factors on individual self-integration behavior. According to the basic needs theory (Deci and Ryan, 2000), the satisfaction of basic needs can stimulate the strengthening of an individual’s internal motivation or make the external environment more acceptable to individuals. The survey results show that 66.5% of college teachers’ basic psychological needs are not met. Lack of basic needs-level satisfaction is one of the reasons why college teachers leave. For the group of college teachers, when their basic needs are not met, they tend to take two different behaviors: the first is to make their own basic demands to colleges and universities and seek relevant suggestions to solve them; the second is to suppress this basic psychological need, and when the suppression fails, they tend to leave. Even take exit actions. Psychologist Shi En introduced the concept of “contract” into the field of psychology, defining “psychological contract” as that in any organization, there is always a set of unwritten expectations between each member and the manager of the organization and others. This expectation itself is a kind of psychological expectation, which is related to the generation of dimission tendency of college teachers (Wang, 2006).

Although the basic demand theory and psychological contract theory point out that there is a close relationship between colleges and university teachers, the process of resignation tendency cannot be grasped from the mechanism of the two. Therefore, it is necessary to introduce cognitive response theory as a supplement to this theory. Cognitive psychology is a psychological trend that emerged in the West in the mid-1950s. It is a psychological mechanism based on human behavior. Its core is the internal psychological process between input and output. Cognitive response theory has three basic assumptions (http://baike.haosou.com/doc/1845539-1951567). First of all, external information appears in the form of stimulus. Secondly, belief not only controls the reception and processing of environmental information by the cognitive system, but also profoundly affects the interaction between the whole cognitive system and external environmental information (Eysenck and Keane, 2005). Finally, cognitive reactions in turn affect existing beliefs and cognitive structures (Axelrod, 1973). It pushes applied cognitive research to a broader real-life space. Cognitive psychology provides a middle perspective for understanding social problems. There is a “critical period” of 6 months for college teachers’ turnover intention to change into behavior. Cognitive psychology provides a medium perspective for understanding the dimission tendency of university teachers, which can be cut into the dimission problem of university teachers from the process dimension (Wang et al., 2007).

## Data and Interview Analysis of Teacher Resignation in Colleges and Universities

### Research Objectives of Data and Interview Research

This study focuses on the problem of college teachers’ turnover tendency. Through the exploration of college teachers’ turnover tendency and psychological mechanism, it establishes a questionnaire on the working status of college teachers to collect data on college teachers’ treatment of work and turnover propensity, and builds a regression model equation to affect the emotional and psychological tendency of college teachers.

### Quantitative Analysis

This survey focuses on the psychological mechanism of college teachers’ resignation tendency and grasps the internal and external causes, occurrence process and willingness direction of the resignation tendency of interviewees through the prevention of questionnaires and interviews, so as to provide data support for the study of countermeasures.

### Data Collection and Analysis Program

To have an overall understanding of the resignation tendency of college teachers, in the process of this survey, we used questionnaires and interviews to evaluate them separately. We randomly selected 6 colleges and universities, randomly selected 4 colleges in each school, distributed 30 questionnaires, each school randomly selected 3 functional departments, and each functional department issued 20 questionnaires, a total of 1,080 questionnaires were distributed, 985 valid questionnaires were recovered, and the effective recovery rate was 91.2%.

This study chose the Rickett Scale compiled by American psychologist Rickett as a research tool to measure the psychological feelings of college teachers in five dimensions, namely, subjective feelings of work pressure, psychological pressure at work, physical pressure at work, positive emotions at work, and negative emotions at work. The scale is mainly composed of a set of statements, including five evaluation indicators, namely, “very little of,” “little of,” “general,” “a little of,” and “much of,” and adopts a five-level scoring system, from 1 point for “very little of,” 5 for “much of” to score five evaluation indicators, and get the psychological feeling of college teachers.

### A Multiple Regression Model for Investigating the Resignation Psychology of the Interviewed Teachers

The SPSS23 software was used to establish a multiple linear regression model of the resignation psychology of the interviewed college teachers through the data collected in the questionnaire from the five dimensions of college work pressure, psychological pressure at work, physical pressure at work, positive emotion, and negative emotion at work, as well as the resignation thought data of teachers, so as to explore the influencing variables of college teachers’ turnover psychology and establish the multiple linear regression model of the resignation psychology of the interviewed college teachers. The specific measurement indicators and various coefficients of the model are shown in Tables 1, 2.

**TABLE 1:** Multiple linear regression model and measurement indicators of resignation psychology of interviewed college teachers.

Model	R	R square	Adjusted R-square	Error of standard estimation	Debin Watson parameter
Multiple regression model of resignation psychology of interviewed College Teachers	0.891	0.794	0.789	0.3866	2.003

**TABLE 2:** Coefficients of multiple linear regression model of resignation psychology of interviewed college teachers.

Multiple regression model		Non-standardized coefficient		Standardization coefficient	t	Significance	Collinearity statistics	
		B	Standard error	Beta			Tolerance	VIF
Dependent variable: Resignation emotion	Argument (constant)	4.242	0.298		14.221	0.000		
	Independent variable: work pressure in Colleges and Universities	0.165	0.052	0.167	3.183	0.002	0.390	2.563
	Independent variable: psychological stress at work	−0.063	0.075	−0.059	−0.841	0.401	0.215	4.641
	Independent variable: physical stress at work	0.133	0.059	0.110	2.263	0.025	0.459	2.180
	Independent variable: positive emotion of doing things	−0.654	0.044	−0.761	−14.882	0.000	0.412	2.426
	Independent variable: negative emotion of doing things	0.003	0.052	0.002	0.065	0.948	0.861	1.161

According to the results of the multiple linear regression model calculated by the SPSS23 software, the adjusted *R*-value in Table 1 is 0.789, which is 0.3 higher than the standard data. Therefore, it is explained that the probability of leaving the university teachers is 78.9%. It is caused by work pressure in college, psychological stress at work, physical stress at work, the positive and negative emotions of doing things, and the Debin Watson parameter in Table 1 is close to 2, so it shows that there is no sequential correlation between various variables.

**FIGURE 1:**
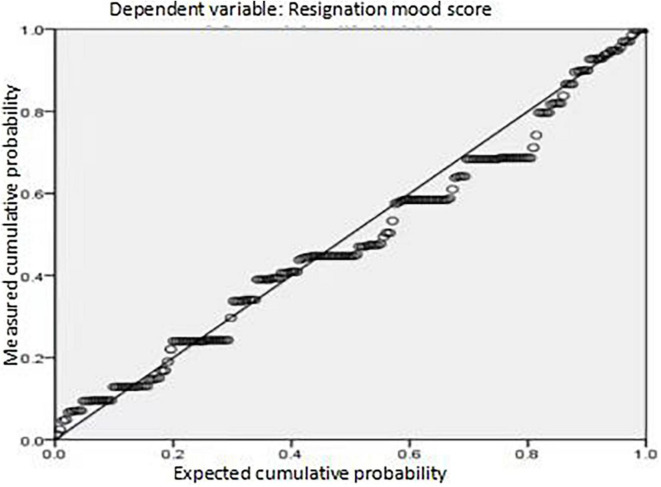
Normal P-P diagram of standardized residuals of resignation psychological regression of interviewed college teachers.

In Table 2, the significance of work pressure, psychological pressure at work, physical pressure at work, positive emotion, and negative emotion in colleges and universities is 0.002, 0.401, 0.025, 0.000, and 0.948, respectively. Among them, the significance of work pressure, physical pressure, and positive emotion in colleges and universities is less than 0.05, so it shows that the physical pressure at work and positive emotions can significantly affect the turnover thoughts of college teachers. The VIF values of collinearity statistics in Table 2 are less than 5, so it shows that there is no multicollinearity in all independent variables. Finally, the normal P-P diagram of the multiple regression model obeys the requirements of normal distribution, and the data are distributed on the diagonal (refer to Figure 1 for details).

According to Table 2, the non-standardized coefficient of college work pressure and physical pressure at work is positive, while the positive emotion of doing things is negative. Therefore, it shows that the greater the work pressure of college teachers, the greater the physical pressure at work, and the lower the positive emotion at work, the stronger the resignation idea of college teachers.

Therefore, according to the above analysis, the multiple linear regression model equation of the resignation psychology of the interviewed college teachers is constructed. The specific form of the equation is shown in formula (1):


(1) A=4.242+0.165*B+0.133*C-0.654*D

Among them, A represents the resignation emotion score of college teachers, B represents the stress score of college work, C represents the physical pressure at work, and D represents the positive emotion of doing things.

### Conclusion of the Study on the Resignation Psychology of the Teachers Who Were Interviewed

**FIGURE 2:**
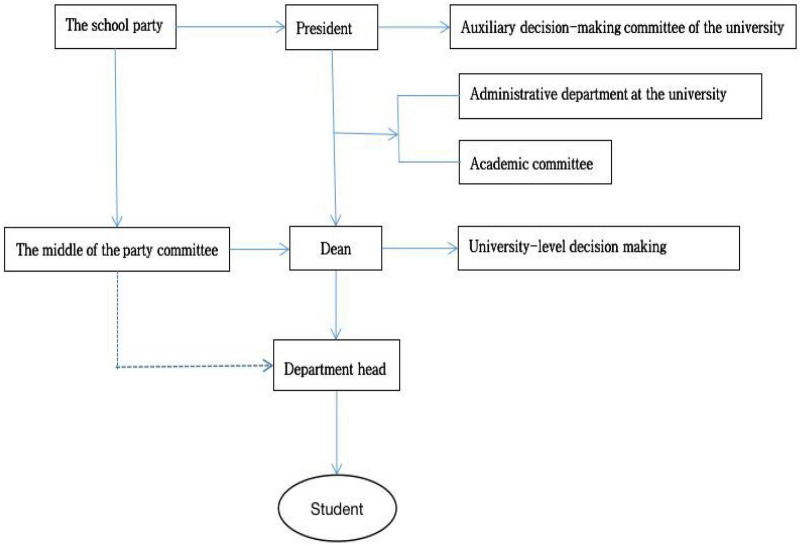
Schematic diagram of university governance structure.

Among the 985 college teachers who were interviewed, 720 have corresponding turnover ideas, accounting for 73%. When external factors such as university performance appraisal and organizational culture as external stimuli exert pressure on the psychology of university teachers, university teachers have more turnover intention (refer to Figure 2 for details).

This feedback produces two results. First, they force themselves to identify with the external environment and adjust their cognitive structure. The specific performance is that although they have turnover intention, they fail to take turnover behavior. Second, use their own cognition to deny the rationality of the external environment. The specific performance is the implementation of resignation action. No matter whether college teachers take resignation behavior or not, the “psychological contract” of college teachers will also disintegrate. The disintegration of psychological contract will enable college teachers to adopt negative strategies to deal with the external pressure such as performance appraisal and organizational culture put forward by colleges and universities, which is the root of the weak cohesion of college talent team.

This study collates the interviews of 35 college teachers among the 985 college teachers interviewed. It is found that the pressure of college teachers mainly comes from three parts. The first part is the teaching pressure. Although college teachers do not need to punch in and go to class on time every day like primary and secondary school teachers, they need to prepare more information about the content of lessons, and also take into account the content that students can accept, and make corresponding adjustments accordingly. During the interview, 23 teachers said that the teachers’ general preparation time is more than three times the class time. With the application of new teaching technologies and methods (such as rain classroom and the teaching integration system represented by wisdom tree), the pressure of lesson preparation is increasingly intensified, which brings great pressure to the daily work of college teachers.

The second part is the pressure of scientific research. Scientific research is the main basis for the evaluation of college teachers’ professional titles. Therefore, college teachers are generally faced with greater pressure of scientific research. Among the 35 college teachers interviewed, 31 are “teaching scientific research” posts. In addition to teaching tasks, they also need to carry out scientific research, and scientific research is the main reference basis for evaluating professional titles. In recent years, the vast majority of colleges and universities have used the performance appraisal mechanism to assess teachers, i.e., if they cannot complete the corresponding workload in a certain period of time (usually 2–3 years) and be rated as deputy senior titles, they will leave. Most of these assessment mechanisms are linked to the establishment, and some colleges and universities no longer provide the newly introduced doctoral establishment. In this environment, scientific research activities greatly increase the psychological pressure of college teachers and reduce their psychological expectations for college work. Then promote them to have turnover intention.

The third part is the pressure on the level of identity. During the interview, 12 teachers mentioned the pressure brought by the school’s organizational culture. In particular, for the newly introduced middle-aged and young teachers, it is difficult to integrate into the organizational culture of the school. The new members are faced with the sense of exclusion brought by the existing social relations at the college level. The existing social relations at the college level often bring psychological pressure to college teachers, which reduces their willingness to integrate into the organizational cultural environment of the university.

Through the follow-up interviews with college teachers, the following basic conclusions are drawn. First, the turnover intention of college teachers is high, but the probability of eventually implementing the turnover behavior is low; second, there is a positive correlation between psychological stress and turnover intention; third, when the gap between the incentives outside colleges and universities and the actual income of college teachers increases significantly, college teachers’ turnover intention is easy to evolve into turnover behavior; and fourth, the work pressure of colleges and universities, the psychological and physical pressure at work, and the emotion of doing things are important reference factors for college teachers’ turnover intention.

## Psychological Analysis of College Teachers’ Turnover Intention

### The Problems of College Teachers’ Resignation

In the process of coping with the external environment created by colleges and universities, the gradually formed psychological contract is an important link to maintain the loyalty of college teachers to colleges and universities. The maintenance of psychological contract itself requires college teachers’ recognition of their working environment and organizational culture. New teachers have more energy to carry out teaching and scientific research. If their efforts cannot be evaluated in a positive way by the school, they will often produce psychological pressure, resulting in physical pressure at work and eventually turnover intention.

Lazarus and Folkman (1984) believed that stress refers to the dynamic process of individual cognitive assessment of the environment. Kyriacou (1987) believed that stress is the bad emotions experienced by teachers in teaching activities, such as tension, frustration, anxiety, and depression. Starting from the concept of pressure, the pressure of college teachers mainly comes from the institutional system formulated by colleges and universities, which leads to the dislocation of college teachers at the role level, e.g., the conflict between the role of teachers in cultural concepts and reality, especially the conflict between the role of teachers in traditional Chinese culture and reality (Liu et al., 2021). In traditional culture, teachers are a role with perfect personality in concept. From Confucius’s “model teacher” to the present “volunteering teacher” image is deeply rooted in the hearts of the people. The virtue of this behavior is the essence of our culture. However, college teachers facing the dual pressure of work and life are usually tired of professional work and lack the sense of gain as teachers. The social relationship of college teachers is relatively simple, the working environment is relatively single, and the work content is relatively boring (Lucy and Helen, 2020). This makes it difficult to coordinate the image expectation of teaching and educating people in the concept with their own work. This dislocation makes many teachers have a sense of disillusionment with lofty ideals. During the discussion, 19 teachers have this sense of disillusionment, and 5 of them think it is difficult for them to dispel this sense of disillusionment. This mismatch between the system and the role of teachers strengthens the psychological pressure of teachers, and when it cannot be adjusted, the dimission behavior is inevitable (Peng, 2021).

### A Scheme for the Rational Flow of College Teachers

First, systematic psychological counseling institutions for college teachers should be established. For college teachers, their turnover intention makes them depressed for a long time. According to the statistics, 84.7% of teachers think that they have a gap between their intention to leave and their real life. In particular, when the personal goals of college teachers are difficult to integrate into the goals of colleges and universities, this depression is more prominent. In the interview, we found that if the respondents highly recognize the values and goals of their unit, even if they have a very strong willingness to leave, they often alleviate their anxiety and pressure through positive ways. Generally speaking, college teachers whose basic psychological needs are met have a strong sense of identity with colleges and universities, and the psychological contract is more stable. Therefore, it is very necessary to take targeted intervention measures to alleviate the psychological pressure of college teachers. Colleges and universities should regularly evaluate the psychological status of teachers. In particular, we should effectively understand the pressure of college teachers through the interview program. In addition to teaching and scientific research, family life, economic pressure, interpersonal relationships, and other problems may be the causes of college teachers’ turnover intention. In particular, there is a lack of systematic mental health counseling institutions for teachers in colleges and universities. Among the six universities selected in our questionnaire survey, only one university will regularly organize psychological lectures for college teachers. In the interview, we found that the turnover intention of teachers in this university is low, and the teachers with turnover intention are also more active. Therefore, colleges and universities should actively establish systematic psychological counseling institutions in colleges and universities. This psychological counseling institution can be led by trade unions, hold regular lectures, and carry out psychological counseling and treatment for individual college teachers.

Second, we should adjust the governance structure of colleges and universities to provide a better external environment for the mitigation of college teachers’ turnover intention. The governance structure of colleges and universities in China is generally shown in the figure above. This hierarchical management structure has the advantages of perfect organization and convenient management, but at the same time, it also brings the problems of complex institutions and large tail. The complexity of university governance structure makes many teachers often tired and at a loss when dealing with schools. To reduce the turnover intention of college teachers, we should adjust the governance structure of colleges and universities and further improve the functions of colleges and universities in serving college teachers, so as to enhance teachers’ identity with the organizational culture of colleges and universities. To alleviate teachers’ turnover intention, the governance structure of colleges and universities can be adjusted for specific functions. The basic idea is to further enhance the service function and reduce (weaken) the examination and approval function. The service-oriented institutions for teachers can establish joint office institutions to facilitate teachers’ work, shorten the work process, and then improve efficiency. For example, we can draw up the staff of the security department, the financial department and the personnel department, and form a comprehensive service department. Teachers need to solve the registered residence problems and financial problems brought about by personnel mobilization, and we can get one-stop solution.

Third, the standards of teacher performance appraisal should be optimized from the school level. Through investigation, we found that a core consideration of current teachers’ dimission is the excessive pressure of performance appraisal. On the one hand, the pressure beyond the norm is not conducive to the maintenance of psychological contract between teachers and schools, and on the other hand, it greatly restrains the efforts of colleges and universities to establish stable teachers. If appropriate adjustment can be made to reduce the quantity standard and improve the quality index, it will be beneficial to restrain the dimission tendency of teachers.

## Conclusion

The turnover intention of college teachers is an objective (increased generally) phenomenon. On the one hand, turnover emotional tendency brings psychological pressure and even physical discomfort to college teachers, which makes it difficult for them to devote themselves to the work process, and then hinders the improvement of work efficiency. On the other hand, the reduction of college teachers’ psychological expectations of colleges and universities is very easy to cause the disintegration of the “psychological contract” between them and colleges and universities, thus affecting the construction of talent team in colleges and universities. Therefore, for the whole academic ecology, we should strengthen efforts to meet the basic psychological and material needs of college teachers and actively promote the rational flow of college teachers, including professional titles, staffing, appropriate teaching, and scientific research tasks. The university institutions should actively take measures to pay attention to the pressure and positive emotion of teachers at work, and build professional psychological counseling institutions for college teachers to help college teachers relieve personal work pressure and physical pressure, so as to make college teachers have positive emotions to face challenges in their work.

## Future Research Directions

To ensure the effectiveness of the data, this study adopts the combination of questionnaire survey and in-depth interview, but the sample is less than the survey object itself, although it is objectively enough to reflect the internal mechanism affecting the resignation of college teachers and provide targeted solutions. However, in the regression analysis to explore the specific weight of each element, the result is not very ideal.

In the future, this research will conduct more in-depth and extensive investigation, and build a more reliable model to demonstrate the internal mechanism of college teachers’ turnover intention on the basis of the current research.

## Data Availability Statement

The original contributions presented in this study are included in the article/supplementary material, further inquiries can be directed to the corresponding author.

## Ethics Statement

Ethical review and approval was not required for the study on human participants in accordance with the local legislation and institutional requirements. Written informed consent from the participants was not required to participate in this study in accordance with the national legislation and the institutional requirements.

## Author Contributions

YZ: propose the research topic, design the research proposal, implement the research process, and draft the thesis. KZ: collect and organize data, research and organize literature, design thesis framework, and revise thesis. Both authors contributed to the article and approved the submitted version.

## Conflict of Interest

The authors declare that the research was conducted in the absence of any commercial or financial relationships that could be construed as a potential conflict of interest.

## Publisher’s Note

All claims expressed in this article are solely those of the authors and do not necessarily represent those of their affiliated organizations, or those of the publisher, the editors and the reviewers. Any product that may be evaluated in this article, or claim that may be made by its manufacturer, is not guaranteed or endorsed by the publisher.

## References

[B1] Axelrod R. (1973). Schema Theory:An Information Processing Model of Perception and Cognition. *J. Am. Po-litical Sci. Rev.* 67 1248–1266. 10.2307/1956546

[B2] Deci E. L. Ryan R. M. (2000). The“what”and“why”of goal pursuits:Human needs and the self-determination of behavior. *Psy-chol. Inq.* 11 227–268. 10.1207/S15327965PLI1104_01

[B3] Du Q. Liu X. J. (2019). The Role of Turnover Tendency and Academic Power Perception of College teachers: an empirical analysis based on the 2016 National College Teacher Development Survey. *J. China High. Educ. Res.* 9 48–53.

[B4] Eysenck M. W. Keane M. T. (2005). *Cognition and Emotion.* Oxford: Oxford University Press.

[B5] Kyriacou C. (1987). Teacher stress and burnout:an international review. *J. Educ. Res.* 29 89–96. 10.1080/0013188870290207

[B6] Lazarus R. S. Folkman S. (1984). *Stress, Appraisal and Coping.* New York: Springer Publishing Company.

[B7] Liu F. Chen H. R. Xu J. Wen Y. Fang T. T. (2021). Exploring the Relationships between Resilience and Turnover Intention in Chinese High School Teachers: Considering the Moderating Role of Job Burnout. *J. Int. J. Env. Res. Public Health* 18 6418–6418. 10.3390/IJERPH18126418 34199322 PMC8296230

[B8] Lucy C. S. Helen F. L. (2020). The Hidden Costs of Teacher Turnover. *J. AERA Open* 6:233285842090581. 10.1177/2332858420905812

[B9] Pei C. X. (2004). On the process control of preventing the loss of teachers in colleges and universities. *J. Hun. Soc. Sci.* 6, 21–24.

[B10] Peng R. X. (2021). Research on the Influence of University Teachers’ Mental Health on Professional Competence. *J. World Sci. Res. J.* 7 244–250. 10.6911/WSRJ.202107_7(7).0033

[B12] Wang M. (2006). Construct the “psychological contract” mode in the management of college teachers in China. *J. J. Changchun University Technol.* 1 63–65.

[B13] Wang T. Z. Zhang F. Q. Fang J. M. (2007). Transfer of Applied Cognition of Modern Cognitive Psychology. *J. J. Shaanxi Normal University* 4 124–128.

